# Btg2 Promotes Focal Segmental Glomerulosclerosis via Smad3‐Dependent Podocyte‐Mesenchymal Transition

**DOI:** 10.1002/advs.202304360

**Published:** 2023-09-25

**Authors:** Qiong‐ Dan Hu, Hong‐Lian Wang, Jian Liu, Tao He, Rui‐Zhi Tan, Qiong Zhang, Hong‐Wei Su, Fahsai Kantawong, Hui‐Yao Lan, Li Wang

**Affiliations:** ^1^ Research Center of Integrated Traditional Chinese and Western Medicine the Affiliated Traditional Chinese Medicine Hospital of Southwest Medical University Sichuan 646000 China; ^2^ Department of Medical Technology Faculty of Associated Medical Sciences Chiang Mai University Chiang Mai 50200 Thailand; ^3^ Department of Nephrology the Affiliated Traditional Chinese Medicine Hospital of Southwest Medical University Sichuan 646000 China; ^4^ Institute of Integrated Chinese and Western Medicine Southwest Medical University Luzhou 646000 China; ^5^ Department of Nephrology the Affiliated Hospital of Southwest Medical University Sichuan 646000 China; ^6^ Cancer Medicine Institute College of Basic Medical Sciences Southwest Medical University Sichuan 646000 China; ^7^ Department of Urology the Affiliated Hospital of Southwest Medical University Sichuan 646000 China; ^8^ Department of Medicine and Therapeutics and Li Ka Shing Institute of Health Sciences the Chinese University of Hong Kong Hong Kong 999077 China

**Keywords:** Btg2, epithelial‐mesenchymal transition, focal segmental glomerulosclerosis, podocyte, TGF‐β/Smad3 signaling

## Abstract

Podocyte injury plays a critical role in the progression of focal segmental glomerulosclerosis (FSGS). Here, it is reported that B‐cell translocation gene 2 (Btg2) promotes Adriamycin (ADR)‐induced FSGS via Smad3‐dependent podocyte‐mesenchymal transition. It is found that in FSGS patients and animal models, Btg2 is markedly upregulated by podocytes and correlated with progressive renal injury. Podocyte‐specific deletion of Btg2 protected against the onset of proteinuria and glomerulosclerosis in ADR‐treated mice along with inhibition of EMT markers such as α‐SMA and vimentin while restoring epithelial marker E‐cadherin. In cultured MPC5 podocytes, overexpression of Btg2 largely promoted ADR and TGF‐β1‐induced EMT and fibrosis, which is further enhanced by overexpressing Btg2 but blocked by disrupting Btg2. Mechanistically, Btg2 is rapidly induced by TGF‐β1 and then bound Smad3 but not Smad2 to promote Smad3 signaling and podocyte EMT, which is again exacerbated by overexpressing Btg2 but blocked by deleting Btg2 in MPC5 podocytes. Interestingly, blockade of Smad3 signaling with a Smad3 inhibitor SIS3 is also capable of inhibiting Btg2 expression and Btg2‐mediated podocyte EMT, revealing a TGF‐β/Smad3‐Btg2 circuit mechanism in Btg2‐mediated podocyte injury in FSGS. In conclusion, Btg2 is pathogenic in FSGS and promotes podocyte injury via a Smad3‐dependent EMT pathway.

## Graphical abstract

Btg2's role in FSGS: Pathogenic via EMT‐induced podocyte injury. Podocyte EMT is enhanced by overexpressing Btg2 but inhibited by specifically deleting Btg2. Furthermore, it uncovered that TGF‐β1 rapidly induces Btg2 and promotes podocyte EMT via a Smad3‐dependent mechanism. Results from this study suggest that targeting Btg2 may be a novel strategy for podocyte injury in FSGS.

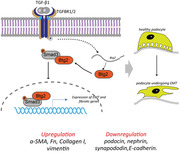

## 1 Introduction

Chronic kidney disease (CKD) is a global public health concern with continuously increased prevalence, resulting in an elevated incidence of end‐stage renal disease (ESRD).^[^ ^1^, ^2^ ^]^ Focal segmental glomerulosclerosis (FSGS) is the most common primary glomerular lesion associated with high‐grade proteinuria and poor prognosis for ESRD.^[^ ^3^, ^4^ ^]^ FSGS is tightly linked with podocyte injury.^[^ ^5^, ^6^ ^]^ Emerging evidence indicates that podocyte epithelial‐mesenchymal transition (EMT) may be a cause of glomerular fibrosis in FSGS.^[^ ^7^, ^8^ ^]^ Podocyte EMT is featured by losing the expression of podocyte‐specific markers such as podocin, synaptopodin, CD2‐associated protein (CD2AP), and the epithelial marker E‐cadherin while acquiring the expression of mesenchymal markers such as vimentin, α‐smooth muscle actin (α‐SMA), type I collagen (Col‐I), type IV collagen (Col‐IV), fibronectin (Fn).^[^ ^9^, ^10^ ^]^ Although podocyte EMT plays a critical role in the pathogenesis of FSGS, mechanisms that mediate podocyte EMT remain unclear, and treatment for FSGS by specifically targeting podocyte EMT remains to be developed.

Transforming growth factor β1 (TGF‐β1) plays a significant role in EMT in various cell types including the podocyte.^[^ ^11^ ^]^ TGF‐β1 treatment decreases the expression of epithelial markers such as nephrin and zonula occludens‐1 (ZO‐1), while it increases the mesenchymal markers including Fn, vimentin, and α‐SMA in the podocytes.^[^ ^12^ ^]^ Furthermore, it is reported that TGF‐β1 induces α‐SMA expression in podocytes through the Smad2/3 pathway.^[^ ^13^ ^]^ On the hand, inhibition of TGF‐β/Smad signaling blocks podocyte EMT and ameliorates albuminuria in diabetic nephropathy.^[^ ^14^, ^15^ ^]^ Thus, TGF‐β/Smad signaling is closely involved in podocyte EMT. However, the mechanism underlying TGF‐β/Smad signaling‐regulated podocyte EMT remains largely unclear.

B‐cell translocation gene 2 (*BTG2*), also known as *PC3* or *TIS21*, belongs to the anti‐proliferation gene family and is the first gene identified in the BTG/TOB family.^[^ ^16^ ^]^ BTG2 is expressed in the spleen, thymus, lung, stomach, large intestine, kidney, etc.^[^ ^17^, ^18^, ^19^ ^]^ BTG2 is a tumor suppressor responsible for cell differentiation, proliferation, apoptosis, and other cellular functions.^[^ ^20^, ^21^ ^]^ It is reported that BTG2 promotes lung and prostate cancer by inducing tumor‐associated EMT.^[^ ^22^, ^23^ ^]^ In addition, *Btg2* mutation is also associated with impaired blood pressure control and renal injury.^[^ ^24^ ^]^ However, the exact role of BTG2 in CKD remains to be elusive.

In rodents, Adriamycin (ADR) can induce rapid podocyte injury featured with massive foot process effacement and glomerulosclerosis, which serves as a model of FSGS.^[^ ^25^, ^26^ ^]^ In the present study, we investigated *Btg2* expression and its role in ADR‐induced FSGS by podocyte‐specifically deleting *Btg2*. Our findings revealed that Btg2 was a pathogenic factor that mediates FSGS via TGF‐β /Smad3‐dependent podocyte EMT.

## 2 Results

### 2.1 Btg2 is Upregulated in Podocytes in FSGS Patients and ADR‐Induced FSGS Mice

**Figure 1:**
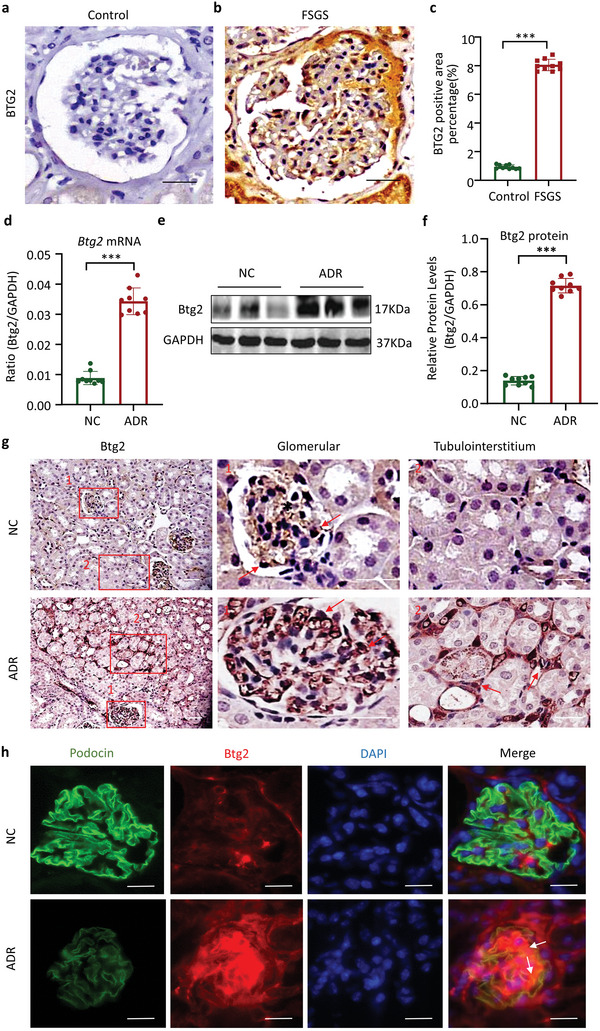
Expression of Btg2 is upregulated in podocytes in FSGS patients and ADR‐induced FSGS mice. a,b) BTG2 immunohistochemical staining. Scale bar = 50 µm. c) Semi‐quantitation of BTG2 expression by immunohistochemical staining. Data are expressed as the mean ± SD (*n* = 10). d) *Btg2* mRNA expression in ADR‐induced nephropathy by real‐time PCR. Data are expressed as the mean ± SD (*n* = 9). e) Western blot analysis of Btg2 in ADR‐induced mice. f) Quantitative analysis of Btg2 protein expression of western blots. Data are expressed as the mean ± SD (*n* = 9). g) Btg2 immunohistochemical staining in ADR‐induced nephropathy. Scale bar = 50 µm. h) Two‐color immunofluorescence shows co‐localization of podocin and Btg2 (arrows) in normal and ADR‐induced FSGS. Scale bar = 50 µm.***P < 0.001 as indicated; NC, normal control; ADR, Adriamycin.

First, we examined Btg2 expression in renal biopsy tissues from patients with FSGS by immunohistochemistry and found that Btg2 expression was up‐regulated in the glomeruli of FSGS patients, presumably by podocytes, compared to control kidney tissues obtained from para‐ renal carcinoma (**Figure** **1**a–c). We then also examined Btg2 expression in a murine FSGS model induced by intravenous injection of 10 mg kg^−1^ ADR in C57BL/6 mice. Western blotting, immunostaining, and RT‐PCR detected that after 8‐week post ADR injection, all mice developed FSGS by significantly increasing urine albumin excretion, glomerular atrophy, and focal segmental glomerulosclerosis (Figure S1a,b, Supporting Information), and a significant loss of podocyte markers podocin and nephrin (Figure S1c–g, Supporting Information). Furthermore, substantial podocyte foot process effacement or even nude glomerular basement membrane was also observed in glomeruli of ADR‐treated mice (Figure S1h, Supporting Information), indicating a significant podocyte injury in a mouse model of ADR‐induced FSGS. We next examined Btg2 expression in ADR nephropathy and detected a marked increase in expression of *Btg2* at both mRNA and protein levels (Figure 1d–f). Immunohistochemistry revealed that compared to a weak Btg2 expression in the control kidney, robust Btg2 expression was observed in the ADR‐treated kidney, presumably by podocytes as well as focally infiltrating cells in the tubulointerstitium (Figure 1g). Notably, double immunofluorescent staining also detected that although ADR induced a loss of podocin, it co‐localized with a marked expression of Btg2 (Figure 1h), suggesting Btg2 may play a role in podocyte injury in ADR‐induced FSGS.

### 2.2 Podocyte‐Specific Deletion of Btg2 Protects against ADR‐Induced FSGS

**Figure 2:**
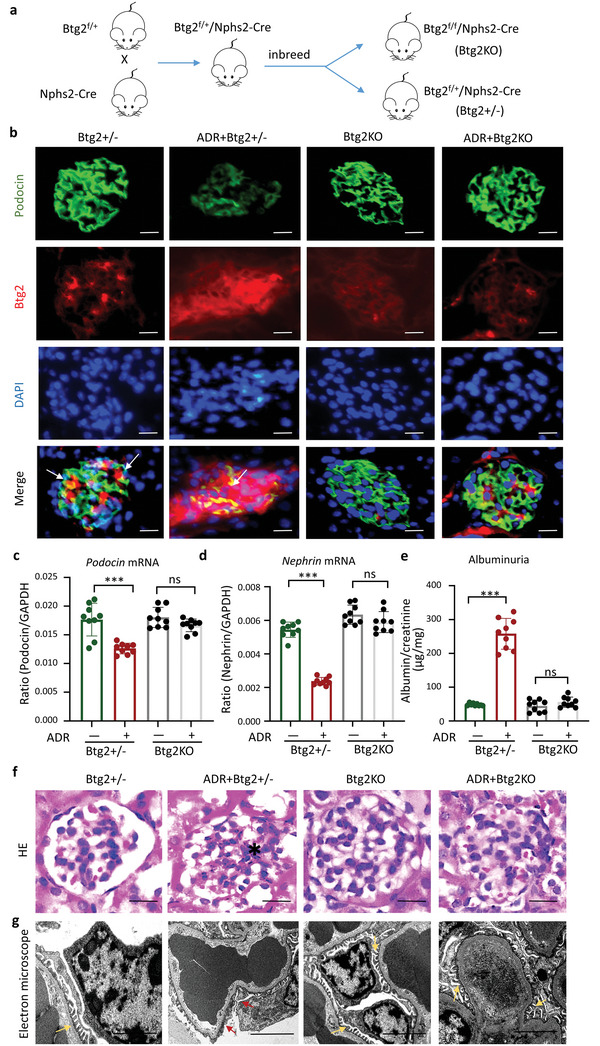
Podocyte‐specific deletion of Btg2 protects against ADR‐induced nephropathy in mice. a) Schematic diagram for generating podocyte‐specific Btg2 knockout mice (Btg2KO). b) Two‐color immunofluorescence for colocalization of podocin and Btg2 (white arrows) in Btg2KO and Btg2^+/‐^ mice with or without ADR injection. Scale bar = 50 µm. c,d) Expression of *podocin* and *nephrin* mRNA determined by RT‐qPCR, Data are expressed as the mean ± SD (*n* = 9). e) ELISA determines the ratio of urinary albumin to urinary creatinine (*n* = 9). f) H&E staining. The black asterisk represents typical focal segmental sclerosis in the glomeruli. Scale bar = 50 µm. g) Electron microscope. The yellow arrows indicate normal podocytes, and the red arrows indicate podocyte detachment, podocyte foot process effacement. Scale bar = 2 µm. ***P < 0.001; ADR, Adriamycin.

To investigate the role of Btg2 in ADR‐induced FSGS, we generated the podocyte‐specific Btg2 knockout mice by crossing the loxp‐flanked *Btg2* mice (*Btg2* ^f/f^) with the Nphs2‐Cre mice in which Cre recombinase is exclusively expressed in the podocyte (**Figure** **2**a). As expected, the offspring *Btg2* ^f/f^/Nphs2‐Cre (Btg2KO) mice showed absence in expression of Btg2 protein in podocytes as revealed by co‐immunofluorescent staining of Btg2 and podocin, whereas *Btg2* ^+/+^/Nphs2‐Cre (Btg2+/‐) mice showed a robust expression of Btg2 upon the ADR induction (Figure 2b). Interestingly, two‐color immunofluorescence revealed that ADR treatment largely reduced podocin expression in glomeruli of the Btg2^+/‐^ mice, which, however, was protected in Btg2KO mice (Figure 2b). This was confirmed at the mRNA levels by real‐time PCR in which ADR‐induced loss of podocin and nephrin were also protected in Btg2KO mice (Figure 2c,d). Furthermore, ADR‐induced albuminuria, glomerular sclerosis, and mesangial expansion in Btg2^+/−^ mice were also inhibited in Btg2KO mice (Figure 2e,f). Moreover, electron microscope revealed that ADR‐induced podocyte injury such as podocyte foot process effacement and detachment as seen in Btg2^+/−^ mice was absent in Btg2KO mice (Figure 2g).

**Figure 3:**
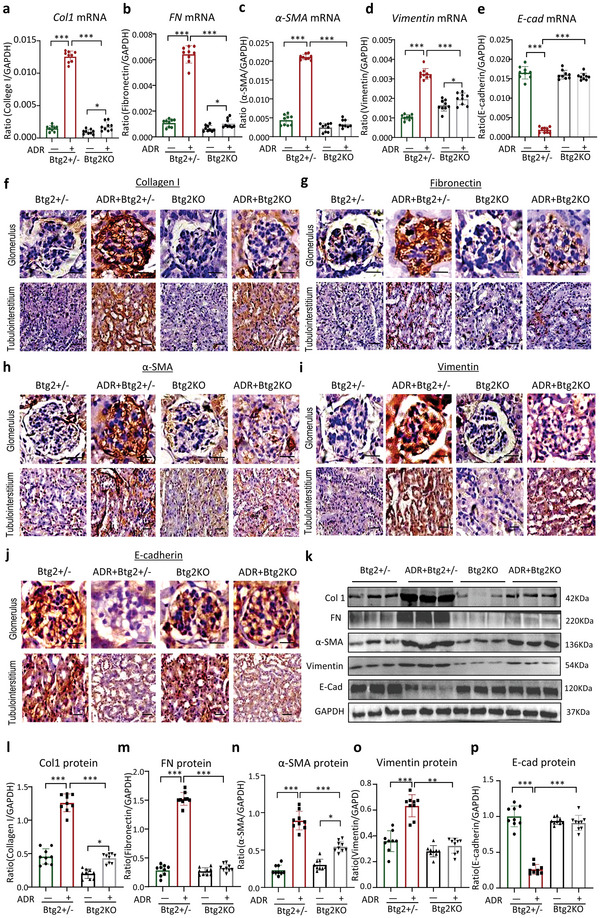
Podocyte‐specific deletion of Btg2 inhibits the podocyte epithelial‐mesenchymal transition (EMT) in mice with ADR‐induced nephropathy. a–e) Real‐time PCR detects mRNA levels of *Collagen1*, *fibronectin, α‐SMA, vimentin*, and *E‐cadherin* in Btg2KO and Btg2^+/‐^ mice, with or without ADR‐induced nephropathy (*n* = 9). f–j) Representative immunohistochemical staining of collagen1, fibronectin, α‐SMA, vimentin, and E‐cadherin in Btg2KO mice and Btg2+/‐ mice with or without ADR injection. Scale bar = 50 µm. k) Western blot analysis of protein levels of collagen1, fibronectin, α‐SMA, vimentin, and E‐cadherin in Btg2KO and Btg2^+/‐^ mice with or without ADR injection. l–p) Quantitative analysis of protein levels detected by western blot analysis. Data are expressed as the mean ± SD (*n* = 9).*P < 0.05, **P < 0.01, ***P < 0.001; ADR, Adriamycin; FN, fibronectin; Vim, vimentin; Col1, collagen1; E‐cad, E‐cadherin.

Glomerular and tubulointerstitial fibrosis is an important feature of FSGS.^[^ ^27^ ^]^ The ADR‐treated Btg2^+/−^ mice showed a marked increase in renal mRNA levels of extracellular matrix genes *Col1a1* and *fibronectin* accompanied by elevated expression of mesenchymal marker genes *α‐SMA* and *vimentin* and decreased expression of epithelial marker protein *E‐cadherin* (**Figure** **3**a–e). By immunostaining and western blotting, ADR‐induced severe glomerular and tubulointerstitial fibrosis as well as EMT as demonstrated by a massive deposition of collagen 1, fibronectin, α‐SMA and vimentin, while decreasing E‐cadherin in Btg2^+/−^ mice were also largely inhibited in Btg2KO mice (Figure 3f–p). To trace the podocyte EMT process, we used a‐SMA as an example to perform two‐color immunofluorescence staining, and the ADR‐treated Btg2+/‐ mice showed elevated expression of mesenchymal marker genes α‐SMA, especially in the parts of the glomerular focal segmental sclerosis where podocin expression was significantly reduced, it is noteworthy that the expression of podocin and α‐SMA is highly overlapping. However, there was no significant change in Btg2KO mice (Figure S2a, Supporting Information).

### 2.3 Btg2 is a Key Mediator in ADR‐Induced Podocyte Injury in Cultured MPC5 Cells

**Figure 4:**
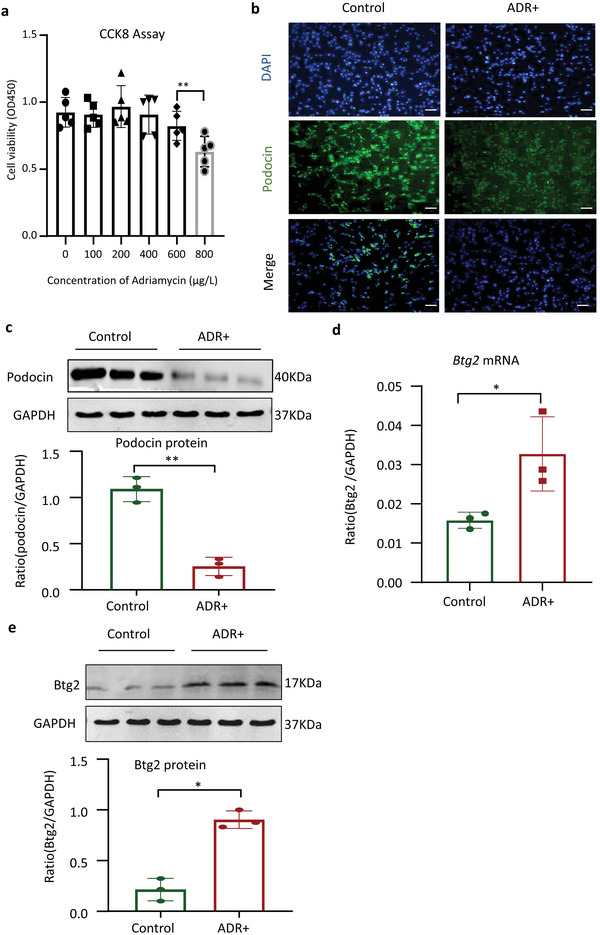
ADR‐induced podocyte injury in cultured MPC5 cells is associated with upregulation of Btg2. a) CCK8 assay to determine the appropriate concentration of ADR in MPC5. Data are expressed as the mean ± SD (*n* = 5). b) Podocin immunofluorescence to evaluate podocyte injury induced by ADR (600 µg L^−1^). Scale bar = 50 µm. c) Western blot analysis of podocin protein levels in response to ADR (*n* = 3). d) *Btg2* mRNA levels determined by RT‐qPCR. Data are expressed as the mean ± SD (*n* = 3). e) The protein levels of Btg2 determined by western blot analysis (*n* = 3). *P < 0.05, **P < 0.01 as indicated, ADR, Adriamycin.

We next examined the regulatory role of Btg2 in ADR‐induced podocyte injury in vitro in a podocyte cell line (MPC5 cells). As determined by CCK8 assay, the MPC5 cell viability was impaired upon treatment with more than 600 ug L^−1^ ADR for 24 h (**Figure** **4**a). By using this dose (600 ug L^−1^), we found that addition of ADR induced a loss of podocyte markers *podocin*, *synaptopodin*, and *CD2AP* (Figure 4b–d; Figure S3a,b, Supporting Information), but markedly upregulated *Btg2* in MPC5 cells (Figure 4e).

**Figure 5:**
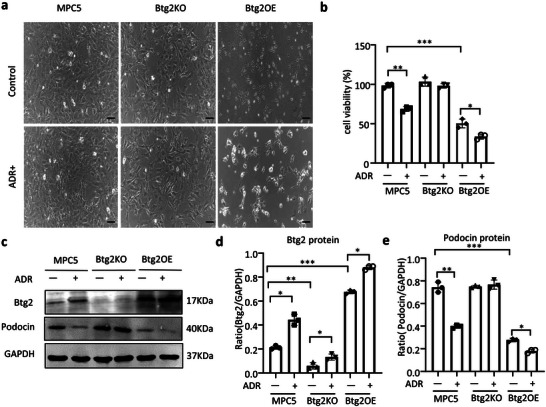
In vitro ADR‐induced podocyte injury in cultured MPC5 cells is further enhanced by overexpressing Btg2 but blocked by disrupting Btg2. a) Cell morphology in response to ADR (600 µg L^−1^) in Btg2 knockout (Btg2KO) or stable Btg2‐overexpressing (Btg2OE) MPC5 cells. b) The CCK8 test to determine the cell viability in MPC5 cells with or without overexpressing or disrupting Btg2 gene in the presence or absence of ADR (600 µg L^−1^). c–e) Western blot and quantitative analysis of Btg2 and podocin expression by MPC5 cells with or without overexpressing or disrupting Btg2 gene in response to the ADR (600 µg L^−1^). Data are expressed as the mean ± SD (*n* = 3). *P < 0.05, **P < 0.01, ***P < 0.001; ADR, Adriamycin; Btg2KO, MPC5 cells with Btg2 knockout; Btg2OE, MPC5 cells overexpressing Btg2.

To further investigate the regulatory role of Btg2 in ADR‐induced podocyte injury, we generated a *Btg2* knockout MPC5 cell line (Btg2KO) by CRISPR/Cas9 technology and a stable *Btg2*‐overexpression MPC5 cell line (Btg2OE) by lentivirus. As shown in **Figure** **5**, addition of ADR largely increased Btg2 but significantly decreased cell viability, resulting in a loss of podocin in MPC5 cells. These were further enhanced by overexpressing *Btg2* gene but were blocked by deleting *Btg2*. Similar results were also found by real‐time PCR in which ADR‐induced loss of *synapotopodin* and *CD2AP* in MPC5 cells was further promoted by overexpressing *Btg2* gene but inhibited by deleting *Btg2* (Figure S4a,b, Supporting Information). All these findings revealed a pathogenic role for Btg2 in ADR‐induced podocyte injury.

**Figure 6:**
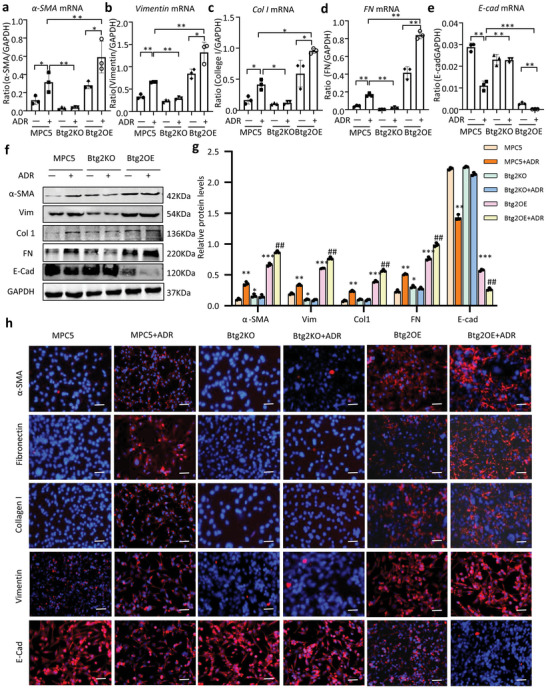
Btg2 promotes ADR‐induced podocyte EMT in cultured MPC5 cells. a–e) Real‐time PCR. f,g) Western blot analysis. h) Two‐color immunofluorescence. Results show that ADR (600 µg L^−1^)‐induced podocyte EMT c at both mRNA and protein levels in MPC5 cells are further enhanced by overexpressing Btg2 but inhibited by disrupting Btg2 gene. Data are expressed as the mean ± SD (*n* = 3). *P < 0.05, **P < 0.01, ***P < 0.001compared with MPC5 cells or as indicated; ^##^ *P* < 0.01, ^###^ *P* < 0.001 compared with MPC5 cells overexpressing Btg2 (Btg2OE). Scale bar = 50µm. ADR, Adriamycin; FN, fibronectin; Vim, vimentin; Col1, collagen1; E‐cad, E‐cadherin.

By using real‐time PCR, western blot, and immunofluorescence, we also found that ADR treatment largely provoked the fibrogenic phenotype of MPC5 cells by increasing α‐SMA and vimentin while decreasing E‐cadherin expression, resulting in EMT with high levels of collagen 1 and fibronectin expression (**Figure** **6**). Surprisingly, ADR‐induced these fibrotic changes in MPC5 cells were exacerbated by overexpressing *Btg2* gene but blocked by deleting *Btg2* (Figure 6). These data suggest that Btg2 plays a pathogenic role in ADR‐induced podocyte injury by promoting the EMT process.

### 2.4 Btg2 Mediates TGF‐β1‐Induced EMT in MPC5 Cells

**Figure 7:**
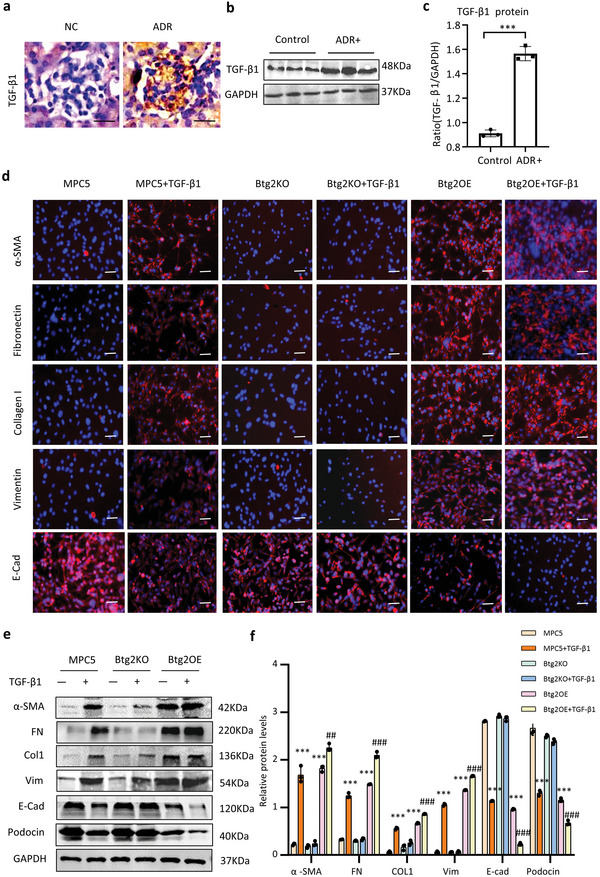
Btg2 promotes TGF‐β1‐induced EMT in MPC5 cells. a)TGF‐β1 immunohistochemical staining in normal and ADR‐induced FSGS mice. Scale bar = 50 µm b,c) Western blot analysis of TGF‐β1 expression in ADR‐induced FSGS mice (*n* = 3). d) Two‐color immunofluorescence reveals that TGF‐β1 (5 ng ml^−1^)‐induced podocyte EMT (increased α‐SMA and vimentin, decreased E‐cadherin) and fibrosis (increased expression of collagen 1 and fibronectin) in MPC5 cells are further enhanced by overexpressing Btg2 (Btg2OE) but inhibited in Btg2KO cells. Scale bar = 50 µm. e,f) Western blot analysis of Protein levels of α‐SMA, fibronectin, Collagen1, vimentin, E‐cadherin, and podocin in TGF‐β‐treated MPC5 cells with or without overexpressing or deleting Btg2. Data are expressed as the mean ± SD (*n* = 3). ***P < 0.001compared with MPC5 cells or as indicated; ^###^ *P* < 0.001compared with Btg2OE cells. ADR, Adriamycin; FN, fibronectin; Vim, vimentin; Col1, collagen1; E‐cad, E‐cadherin.

It has been reported that TGF‐β signaling plays an important role in podocyte EMT associated with FSGS.^[^ ^28^, ^29^ ^]^ In the present study, we also detected TGF‐β1 was upregulated in the glomeruli of ADR nephropathy and ADR‐treated MPC5 cells (**Figure** **7**a–c). Like ADR stimulation, addition of TGF‐β1 also induced podocyte injury and fibrotic EMT phenotype in MPC5 cells as demonstrated by inhibiting expression of podocin, synaptopodin, and CD2AP but increasing expression of α‐SMA, vimentin, collagen 1, and fibronectin while decreasing E‐cadherin expression (Figure 7d–f; Figure S5a–g, Supporting Information). All these fibrotic changes in response to TGF‐β1 were exacerbated in MPC5 cells that are highly overexpressing Btg2 but were blocked in those with Btg2 deletion (Figure 7d–f; Figure S5a–g, Supporting Information).

### 2.5 Btg2 is a Quick Responsive Protein Stimulated by TGF‐β1

**Figure 8:**
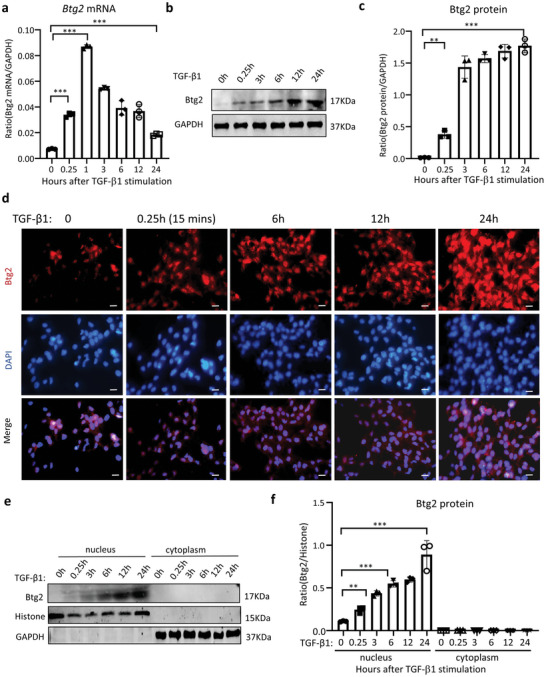
Btg2 is a quick responsive nuclear protein from podocytes (MPC5 cells) stimulated by TGF‐β1. a) Real‐time PCR (*n* = 3). b,c) Western blot (*n* = 3). d) Two‐color immunofluorescence. Results show that addition of TGF‐β1 (5 ng ml^−1^) induces a rapid upregulation of Btg2 mRNA and protein expression by MPC5 cells as early as 15 min. e,f) Western blot analysis shows TGF‐β1‐induced a rapid Btg2 expression in the nucleus but not in cytoplasm of MPC5. Data are expressed as the mean ± SD (*n* = 3).**P < 0.01, ***P < 0.001 as indicated.

Interestingly, in MPC5 cells, TGF‐β1 treatment rapidly upregulated *Btg2* mRNA level as fast as in 15 min, which was culminated at 1 h. At 24 h post TGF‐β1 treatment, *Btg2* mRNA remained high (**Figure** **8**a). These findings suggest that the quick responsiveness of *Btg2* mRNA in response to TGF‐β1 may occur at the post‐transcriptional level. Western blot and immunofluorescence also observed a time‐dependent steady increase of Btg2 protein after TGF‐β1 treatment (Figure 8b–d). Furthermore, by cytoplasm and nuclei separation, we found that TGF‐β1‐induced Btg2 expression was exclusively occurred in the nucleus (Figure 8e,f).

### 2.6 Btg2 Directly Interacts with Smad3 but not Smad2 to Mediate Podocyte EMT via a Smad3‐Dependent Mechanism

**Figure 9:**
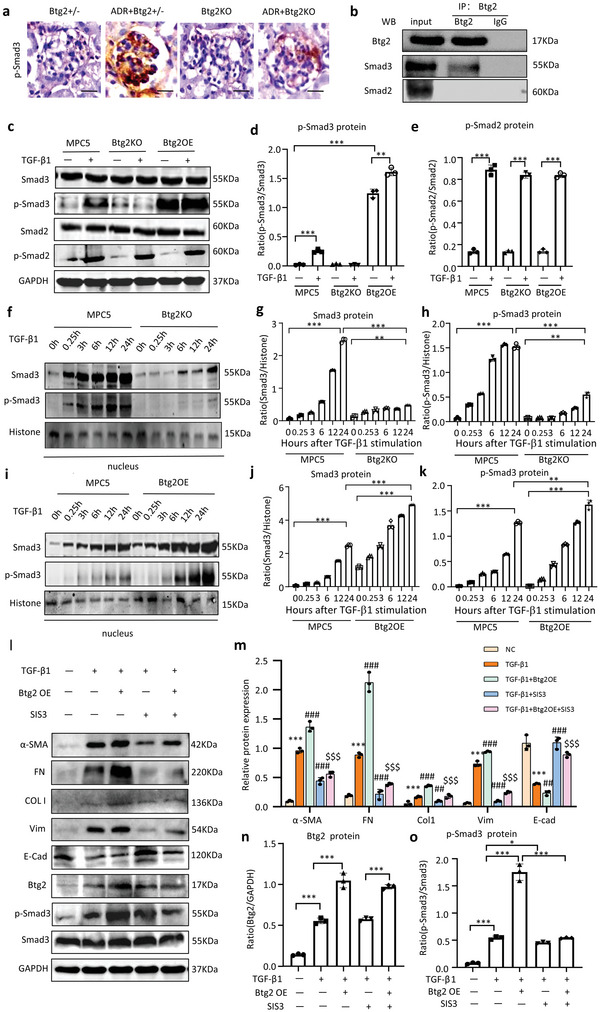
Btg2 directly interacts with Smad3 but not Smad2 to mediate podocyte EMT via a Smad3‐dependent mechanism. a) Mice lacking podocyte Btg2 can protect ADR‐induced p‐Smad3 expression in the glomerulus with FSGS (*n* = 9). Scale bar = 50 µm. b) Co‐immunoprecipitation detects that Btg2 can directly interact with Smad3 but not Smad2. c–e) Western blot analysis shows that TGF‐β1 (5 ng ml^−1^)‐induced activation of Smad3 signaling in MPC5 cells is significantly enhanced by overexpressing Btg2 but blocked by deleting Btg2. In contrast, alteration of Btg2 expression does not influence TGF‐β1‐induced Smad2 phosphorylation (*n* = 3). f–h) Western blot analysis shows that deletion of Btg2 from MPC5 cells abrogates TGF‐β1‐indiced Smad3 and p‐Smad3 nucleated translocation (*n* = 3). i–k) Western blot analysis shows that overexpression of Btg2 in MPC5 cells largely promotes TGF‐β1‐indiced Smad3 and p‐Smad3 nucleated translocation (*n* = 3). l–o) Western blot analysis shows that overexpression of Btg2 significantly promotes TGF‐β1‐induced Smad3 signaling and podocyte EMT/fibrosis, which is blocked by a Smad3 inhibitor SIS3 (*n* = 3). Data are expressed as the mean ± SD. *P < 0.05, ***P < 0.01, ***P < 0.001 compared with control (NC) or as indicated; ^##^ *P* < 0.01, ^###^ *P* < 0.001 compared with TGF‐β1‐treated cells; ^$$$^ *P* < 0.001 compared with TGF‐β1‐treated Btg2OE cells.

We next investigated whether the TGF‐β1‐responsive Btg2 can influence the activity of TGF‐β signaling. We first examined TGF‐β1 expression in ADR‐induced FSGS and found that a massive Smad3 activation (phosphorylation) was evidenced in the glomeruli of ADR‐induced nephropathy (**Figure** **9**a), which was blocked by podocyte‐specific *Btg2* knockout (Figure 9a). It has been reported that Btg2 can interact with Smad1 and Smad8 to mediate the BMP signaling.^[^ ^30^ ^]^ It is also well documented that TGF‐β1 mediates tissue fibrosis and EMT via a Smad3‐dependen mechanism.^[^ ^31^, ^32^ ^]^ We then hypothesized that Btg2 may promote TGF‐β/Smad3 signaling to mediate podocyte injury by physically interacting with Smad3. By co‐immunoprecipitation, Btg2 could interact directly with Smad3 but not Smad2 in MPC5 cells (Figure 9b). Thus, Btg2 overexpression promoted, whereas Btg2 deletion suppressed TGF‐β1‐induced Smad3 phosphorylation without altering phosphorylation levels of Smad2 (Figure 9c–e), suggesting that Btg2 may specifically promote Smad3 signaling.

As Btg2 is located in the nucleus, we further hypothesized that Btg2 may contribute to the nuclear translocation of phosphorylated Smad3. Western blot analysis of the nuclear lysate showed that Btg2 overexpression promoted, whereas Btg2 deletion attenuated TGF‐β1‐stimulated Smad3 nuclear accumulation (Figure 9f–k). Importantly, we also detected that addition of a Smad3 inhibitor SIS3 was capable of inhibiting TGF‐β1‐induced Btg2 expression, the promoter activities of Smad3, and the development of podocyte fibrotic phenotype including EMT (Figure 9l–o). These findings revealed that Btg2 is upregulated by TGF‐β1 and functions to mediate podocyte injury such as EMT and fibrogenesis via a Smad3‐dpendent mechanism.

**Figure 10:**
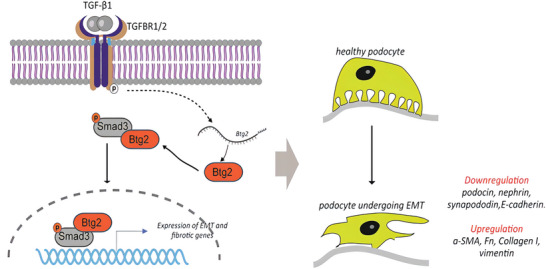
Visual abstract.

In brief, the present study discovered that Btg2 is pathogenic in FSGS and mediates podocyte injury via EMT, which is enhanced by overexpressing Btg2 but inhibited by specifically deleting podocyte Btg2 gene. Furthermore, we uncovered that Btg2 is rapidly induced by TGF‐β1 and promotes podocyte EMT via a Smad3‐dependent mechanism. The results are displayed as a visual abstract (**Figure** **10**).

## 3 Discussion

FSGS is a life‐threatening nephropathy and podocyte injury such as EMT plays a critical role in the disease progress.^[^ ^7^, ^28^, ^33^ ^]^ Although Btg2 mutation induces hypertension and proteinuria in female high‐salt diet‐fed rats, deletion of Btg2 shows no significant phenotype in the mouse kidney.^[^ ^34^, ^35^ ^]^ In this study, we found that Btg2 was significantly upregulated in glomerular cells, especially in podocytes, in patients and mice with FSGS. Importantly, we discovered that Btg2 is pathogenic in FSGS as conditional knockout of podocyte Btg2 protected against ADR‐induced FSGS by preventing podocyte injury such as the loss of podocyte markers, EMT, and the development of proteinuria and progressive glomerulosclerosis. The functional role of Btg2 was further demonstrated in a mouse podocyte cell line MPC5 in which deletion of Btg2 protected against, whereas overexpression of Btg2 promoted ADR and TGF‐β1‐induced podocyte injury including the loss of podocyte markers, EMT and collagen matrix production. Results from this study identified a new role for Btg2 in the pathogenesis of podocyte injury associated with ADR‐induced FSGS.

TGF‐β signaling is the predominant pathway to regulate podocyte EMT and contributes to the disease progress of FSGS.^[^ ^15^ ^]^ However, little knowledge is known on how TGF‐β signaling is modulated during podocyte EMT. In this study, we found that upregulation of podocyte Btg2 was tightly regulated by TGF‐β1. Indeed, addition of TGF‐β1 induced a rapid expression of Btg2 mRNA and protein as early as 15 min. This suggests that TGF‐β1‐regulated expression of Btg2 likely at the post‐transcriptional level. We also revealed that TGF‐β1 induced podocyte Btg2 expression via the Smad3‐dependent mechanism as blockade of Smad3 signaling with a Smad3 inhibitor SIS3 inhibited TGF‐β1‐induced Btg2 expression in MPC5 cells. Moreover, we also revealed that Btg2 could bound directly to Smad3 but not Smad2 to promote Smad3 phosphorylation and nucleated translocation, which was further enhanced by overexpressing Btg2 but blocked by deleting Btg2 in MPC5 cells. These novel findings suggest a TGF‐β/Smad3‐Btg2 circuit mechanism in Btg2‐mediated podocyte injury. This was further supported by the findings that in TGF‐β1‐treated MPC5 cells, overexpression of Btg2 enhanced podocyte EMT and fibrosis, which was blocked by a Smad3 inhibitor. As Smad3 but not Smad2 is the primary effector of TGF‐β signaling to mediate EMT and fibrosis in the epithelial cells,^[^ ^36^, ^37^ ^]^ it is highly possible that Btg2 may orchestrate the downstream TGF‐β/Smad3 signaling to mediate podocyte injury in FSGS.

Podocyte‐mesenchymal transition (PMT) is indeed a relatively new concept in the context of fibrosis,^[^ ^38^ ^]^ and the role of EMT in renal fibrosis is still a subject of debate.^[^ ^39^ ^]^ Several studies have suggested that the contribution of EMT to fibrosis is less than 5%. Therefore, it is important to explore specific methods for tracing podocyte‐mesenchymal transition in the context of FSGS. However, it can be challenging to capture direct evidence of EMT during pathological processes. The reason is it is crucial to consider that the contribution of PMT to FSGS may vary in different individuals and contexts. In present study, expression changes of PMT‐related markers were found in ADR or TGF‐β1‐induced podocyte's injury: demonstrated by increasing α‐SMA and vimentin while decreasing E‐cadherin, and fibrosis by upregulating collagen1and fibronectin. The two‐color immunofluorescence co‐staining of podocin and α‐SMA also manifested the feature of PMT. These results reflect the process of PMT and its contribution to glomerular sclerosis or fibrosis to some extent. However, further research is needed to understand the precise mechanisms and identify reliable tracing methods for PMT in FSGS.

There also exist certain questions left to resolve in current study. First, we still lack the knowledge on Btg2 expression pattern in non‐FSGS podocyte diseases like diabetic nephropathy. In fact, we speculate that Btg2 may be highly expressed and play a role in promoting disease in diabetic nephropathy, membranous nephropathy, and minimal change disease manifested by podocyte injury, which needs to be further confirmed. Except podocytes, endothelial cells, mesangial cells, and macrophages may contribute to the development of FSGS.^[^ ^40^, ^41^, ^42^, ^43^ ^]^ In fact, there are some clues in our research. In the kidneys of ADR‐induced FSGS mice, Btg2 is upregulated in the renal interstitial, which implies that renal interstitial cells such as macrophages may be involved. Therefore, it is necessary to explore the position of Btg2 in other renal intrinsic cells in the future. Besides, we are also unclear about the detailed mechanisms regarding how Btg2 is transiently upregulated upon TGF‐β1 stimulation and mediates the downstream nuclear translocation of Smad3. It is interesting and full of challenges.

In conclusion, the present study demonstrate that Btg2 is a novel mediator in FSGS. Btg2 is induced by TGF‐β1 and mediates podocyte EMT and fibrosis responses via a Smad3‐dependent mechanism. Results from this study suggest that targeting Btg2 may be a promising therapeutic strategy for FSGS.

## 4 Experimental Section

### Human Kidney Samples

Kidney biopsy samples were obtained from 10 patients with diagnosed FSGS in the Affiliated Hospital of Southwest Medical University from 2021 to 2022. Whereas 10 control samples were obtained from para‐tumor tissues in patients who underwent surgical removal of renal carcinoma. Details of patient information are shown in Table S1 (Supporting Information). There was no statistical difference in general information of patients (gender, age) between the two groups. Informed written consent was obtained from all participants or next of kin. The protocol was approved by the Clinical Trial Ethics Committee, Affiliated Hospital of Southwest Medical University, China (reference No. KY 2 022 192).

### Animal Models of ADR‐Induced FSGS in Podocyte Btg2 Knockout Mice

The conditional *Btg2* knockout mouse (*Btg2* ^f/f^, C57BL/6 background) was generated by insertion of the loxp cassettes in the 1^st^ and 2^nd^ intron of *Btg2* genomic locus by using the CRISPR/Cas9‐mediated genomic edition (Cyagen, Guangzhou, China). The Nphs2‐Cre mice were purchased from the Jackson Laboratory (Strain #:0 08205, C57BL/6 background). The *Btg2* ^f/f^ and Nphs2‐Cre mice were crossed to generate the podocyte conditional *Btg2* knockout (Btg2KO) offspring (*Btg2* ^f/f^/Nphs2‐Cre). The littermates with the genotype of *Btg2* ^f/+^/Nphs2‐Cre (Btg2+/‐) were used as control. The wild‐type C57BL/6 mice were purchased from the Gem Pharmatech Co, Ltd (Chengdu, China). All animals (*n* = 9, total 54) were maintained in a specific pathogen‐free facility with free access to food and water. Sample size was decided by previous study^[^ ^44^ ^]^ and testing requirements. The mice were blindly and randomly divided into groups using a table of random numbers. For ADR‐induced FSGS, the mice were injected with 10 mg kg^−1^ ADR (Shenzhen Main Luck Pharmaceuticals Inc, China) via tail vein. The sham‐operated mice were injected with vehicle (saline). Eight weeks post ADR injection, mice were sacrificed by intraperitoneal injection with overdose of pentobarbital sodium followed by collection of blood and kidney for downstream analysis. All mice enrolled for the ADR‐induced FSGS study were male at the age of 8 to 10‐weeks (22–25 g). The animals were monitored by the research unit once daily. If the mice got the following symptoms, including lethargy, shortness of breath, skin discoloration or irregularity, enlargement of lymph nodes, and solid visible tumors under the subcutis, mice were euthanized directly. These morbid mice were documented as “adverse events”. The animal protocol was approved by the Animal Ethics Committee of Southwest Medical University, China (reference No. 20221031‐012). The ARRIVE1 reporting guidelines were used.^[^ ^45^ ^]^

### Urine Albumin‐Creatinine Ratio

The random urine was collected for determination of the concentration of albumin by the mouse albumin enzyme‐linked immunosorbent assay kit (Sangon Biotech, Cat# D721120, China) and creatinine by a creatinine assay kit (Nanjing Jiancheng, Cat# C011‐2‐1, China) according to the manufacturers’ instructions. The urine albumin‐creatinine ratio (UACR) was calculated by dividing the concentration of urine albumin with the concentration of creatinine.

### Hematoxylin‐Eosin Staining

Mouse kidney was fixed in 4% neutral formaldehyde followed by paraffin embedding. The paraffin sections (4 µm) were rehydrated in gradient ethanol and subjected to hematoxylin‐eosin (HE) staining (Beyotime, Shanghai, China) as previously described.^[^ ^46^ ^]^

### Immunohistochemistry

The formalin‐fixed, paraffin sections (4 µm) were treated with 3% H_2_O_2_ to deactivate endogenous peroxidase for 10 min at room temperature (RT), followed by antigen retrieval in 0.01 m citrate solution (pH 6.0) in microwave oven for 10 min. Then, the sections were blocked with 2.5% bovine serum albumin (BSA) for 1 h at RT and incubated with indicated primary antibody at 4 °C overnight. Next, the Universal Mouse/Rabbit HRP‐conjugated Polymer Detection Kit (ZSGB‐Bio, Cat# PV‐6000, China) was used to recognize the primary antibody. Staining signals was visualized by DAB chromogenesis. Nuclei were counterstained with hematoxylin.

### Immunofluorescence

For immunofluorescence, the OCT‐embedded kidney sections (4 µm) were permeabilized in 0.25% Triton X‐100 (in PBS) for 10 min and blocked in 2.5% BSA for 1 h at RT, and then stained with the primary antibody for overnight at 4 °C, followed by fluorescence‐conjugated secondary antibody at RT for 1 h. Sections were counterstained with DAPI.

Antibodies used for immunostaining are listed in Table S2 (Supporting Information). All sections were examined under the Leica DM4B upright digital research microscope (Leica, DMC6200, Germany) and the intensity of staining signals was analyzed by the ImageJ 1.47 V software (Rawak Software Inc., Stuttgart, Germany).

### Cell Culture

The conditionally immortalized mouse podocyte cell line (MPC5) was kindly provided by Prof. San‐Tao Ou (Department of Nephrology of the Southwest Medical University). The cells were cultured in RPMI‐1640 medium supplemented with 10% fetal bovine serum (FBS), and 10 IU mL^−1^ recombinant IFN‐γat 33 °C. To induce differentiation, the MPC5 cells were cultured at 37 °C for 14 days. The differentiated MPC5 cells were then treated with different doses of ADR (0100, 200, 400, 600, 800 µg L^−1^) for 24 h. To examine the regulatory role of TGF‐β/Smad3 signaling in Btg2 expression, after being starved in 0.5% FBS for 12 h, the differentiated MPC5 cells were treated with 5 ng mL^−1^ TGF‐β1 for 0, 0.25, 3, 6, 12, 24 h. The role of Smad3 in TGF‐β1‐induced Btg2 expression was also examined by addition of a Smad3 inhibitor SIS3 (5 µm, SIGMA, S0447, USA) at 30 min before stimulation with TGF‐β1. To study the role and mechanisms of Btg2 in podocyte injury, the *Btg2* knockout MPC5 cell line (Btg2KO) was generated by the Crispr/Cas9 system as previously described.^[^ ^47^ ^]^ Whereas the stable *Btg2‐*overexpressing MPC5 cell line (Btg2OE) was also generated by transfecting the *Btg2‐*overexpressing lentivirus followed by puromycin resistance selection.

### Cell Counting Kit‐8 Assay

MPC5 cells were seeded into 96‐well plates with 5 × 10^3^ cells each well. Next day, the cells were treated with different concentrations of ADR for 24 h as described above. Then, the medium was changed with serum‐free medium containing 10% the Cell Counting Kit‐8 (CCK‐8) reagent (Dojindo, Kumamoto, Japan) followed by incubation for 1 h. The absorbance of 450 nm wavelength was measured by a microplate reader (BioTek, SYNEGY2, USA).

### Western Blot Analysis

Total protein was extracted from the renal cortex or MPC5 cells by RIPA lysis buffer (Beyotime, China). Nuclear and cytoplasmic protein was extracted from MPC5 cells with a commercial kit (Beyotime, P0027, China) following the manufacturer's instruction. Protein concentration was measured by the Bicinchoninic Acid (BCA) Protein Quantitation Kit (Beyotime, P0010S, China). Thirty‐five micrograms protein was separated in 10–15% SDS‐PAGE gel and transferred to the PVDF membrane (Millipore, ISEQ00005, USA). Then, the membrane was blocked with 5% BSA and incubated with indicated antibody at 4 °C overnight. After rinsing, the membrane was incubated with the corresponding HRP‐conjugated secondary antibody at RT for 2 h. The signals were detected with the ChemiDoc TM (Chemiscope 6200, China) or Amersham Typhoon (amTypho, USA) detection system. The gray value of the band was quantitated with the ImageJ 1.47 V software (Rawak Software Inc., Stuttgart, Germany). Antibodies used in this study are listed in Table S2 (Supporting Information).

### RT‐PCR

Trizol reagent (Tiangen, RK145, China) was used to extract total RNA from mouse kidney and MPC5 cells. Reverse transcription was performed using the Reverse Transcription Kit (Promega, A5003, China). Then, PCR was performed with the Universal SYBR qPCR Master Mix (Vazyme, Q711‐02, China) on the LightCycler 480 amplification system (Roche, SN6402, Germany). Primers used in RT‐PCR are listed in Table S3 (Supporting Information). The relative expression of detected genes was normalized to GAPDH by the 2^−ΔΔCt^ method.

### Co‐Immunoprecipitation

Total protein of MPC5 cells was isolated with the RIPA buffer. After clarification by centrifugation, 1 µg mice anti‐Btg2 antibody or its non‐specific isotype immunoglobin was added into 600 µg total cellular protein in a volume of 500 µL. The mixture was incubated with slow rotation at 4 °C overnight. Next day, 20 µL Protein A agarose beads (Beyotime, Cat# P2051, China) were added to the mixture followed by rotation at 4 °C for another 2 h. Then, the agarose beads were spin down and washed with 1 mL RIPA buffer for 4 times. After the final spinning down, 30 µL 1  × SDS loading buffer was added followed by boiling for 5 min to release the protein from the beads for downstream western blotting analysis.

### Statistical Analysis

The statistical tests for the normal distribution of data included the Kolmogorov‐Smirnov test and Shapiro‐Wilk test. The normally distributed data were depicted as the mean ± standard deviation (SD) by scatter plots, and a t‐test or one‐way ANOVA test was applied to compare each group. If data were not normally distributed or variances were unequal, the data were depicted as the median and the first quartile to the third quartile by scatter plots, and the differences between each group were analyzed using the Mann‐Whitney test or the Kruskal‐Wallis test. Statistical analysis was performed with GraphPad Prism 9.0 software (GraphPad Software Inc CA, USA). The *p* value < 0.05 was statistically significant.

### Institutional Review Board Statement

The study was reviewed and approved by the Ethics Review Committee of the Affiliated Traditional Chinese Medicine Hospital of Southwest Medical University (Approval No. 20221031‐012).

## Conflict of Interest

The authors declare no conflict of interest.

## Author Contributions

Q.‐D.H. and H.‐L.W. contributed equally to this work. L.W. conceived and designed the project. F.K. and H.‐Y.L. designed the experiments. Q.‐D.H. and H.‐L.W. performed most of the experiments and collected results. R.‐Z.T. participated in animal experiments. T.H. revised the manuscript. J.L., Q.Z., and H.‐W.S. helped in Clinical data collection. Q.‐D.H., L.W., and H.‐Y.L. provided funding for the project. All authors read the manuscript, provided feedback, and approved the final manuscript.

## Supporting information

Supporting Information

## Data Availability

The data that support the findings of this study are available from the corresponding author upon reasonable request.
