# Supplementary material for: Btg2 Promotes Focal Segmental Glomerulosclerosis via Smad3‐Dependent Podocyte‐Mesenchymal Transition
Source: Adv Sci (Weinh). 2023 Sep 25;10(32):2304360. doi: 10.1002/advs.202304360 (PMC10646233; doi:10.1002/advs.202304360)
Supplement: Supplementary file 1 — Supporting Information [file ADVS-10-2304360-s001.pdf]

ADVANCED SCIENCE
Open Access

Supporting Information

for *Adv. Sci.*, DOI 10.1002/advs.202304360

Btg2 Promotes Focal Segmental Glomerulosclerosis via Smad3-Dependent Podocyte-Mesenchymal Transition

Qiong- Dan Hu, Hong-Lian Wang, Jian Liu, Tao He, Rui-Zhi Tan, Qiong Zhang, Hong-Wei Su, Fahsai Kantawong, Hui-Yao Lan* and Li Wang**

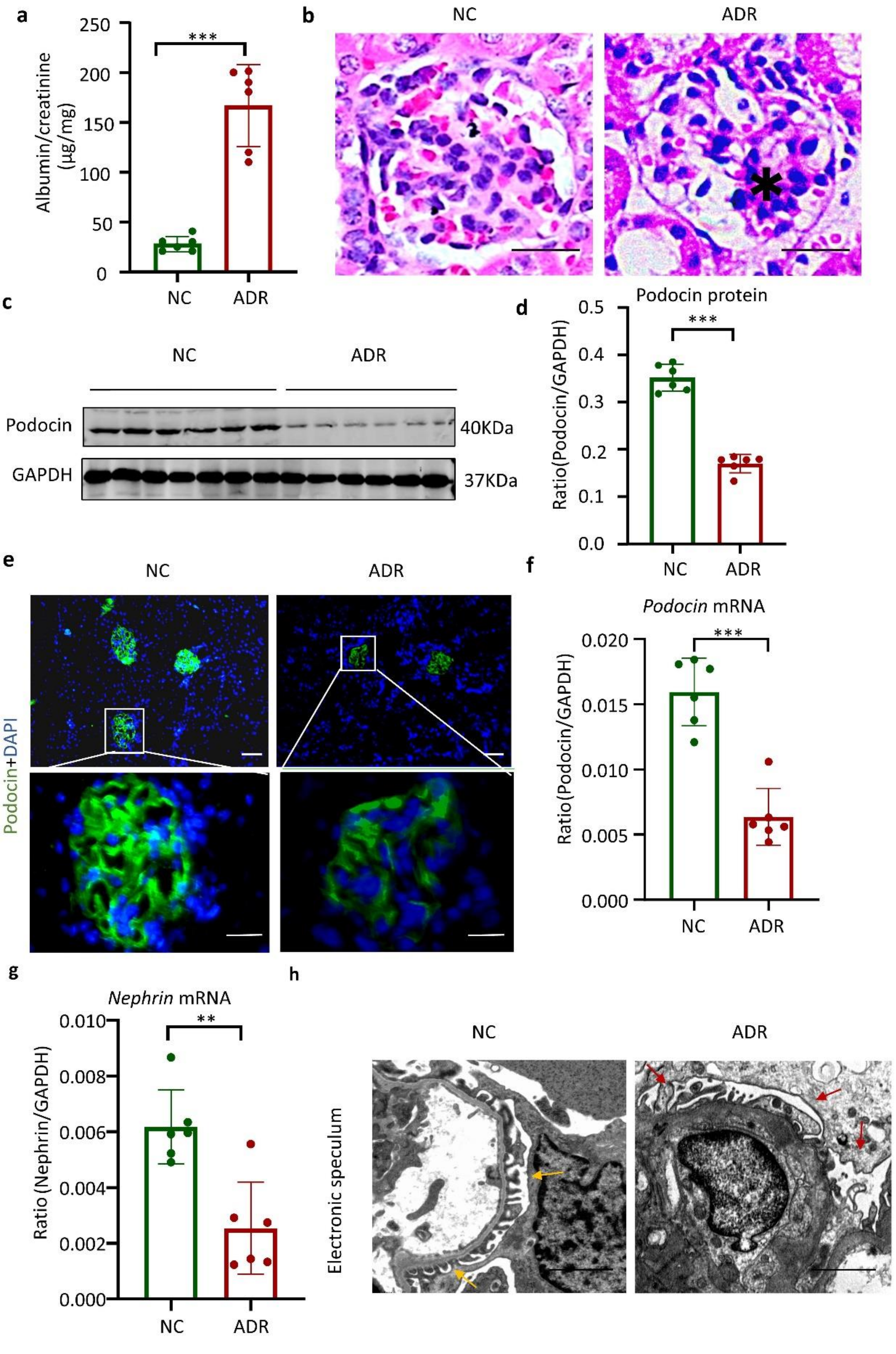

Supplementary Figure 1. The focal segmental glomerulosclerosis (FSGS) model

was established in C57BL/6 mice. (a) ELISA determines the ratio of urinary albumin to urinary creatinine. Data are expressed as the mean±SD(n=6). **(b)**Hematoxylin and eosin (H&E) staining was used to evaluate the pathology after adriamycin (ADR) injection. The black asterisk represents typical focal segmental sclerosis in the glomeruli. Scale bar =50μm. **(c)** The protein levels of podocin after ADR injection was determined by western blotting(n=6). **(d)** Quantified by western blot analysis and normalized to control. Data are expressed as the mean±SD(n=6). **(e)** Podocin immunofluorescence staining was used to evaluate glomerular podocyte injury after ADR injection. Scale bar =50μm. **(f-g)** The mRNA levels of *podocin* and *nephrin* after ADR injection was determined by RT-qPCR. Data are expressed as the mean±SD(n=6). **(h)** Electron microscope was used to evaluate the podocyte injury after ADR injection. The yellow arrow points to normal podocytes, and the red arrow points to abnormal podocytes (Podocyte detachment, podocyte fuse, and effacement). Scale bar =2μm. ***P<0.001. NC, Normal control; ADR, Adriamycin.

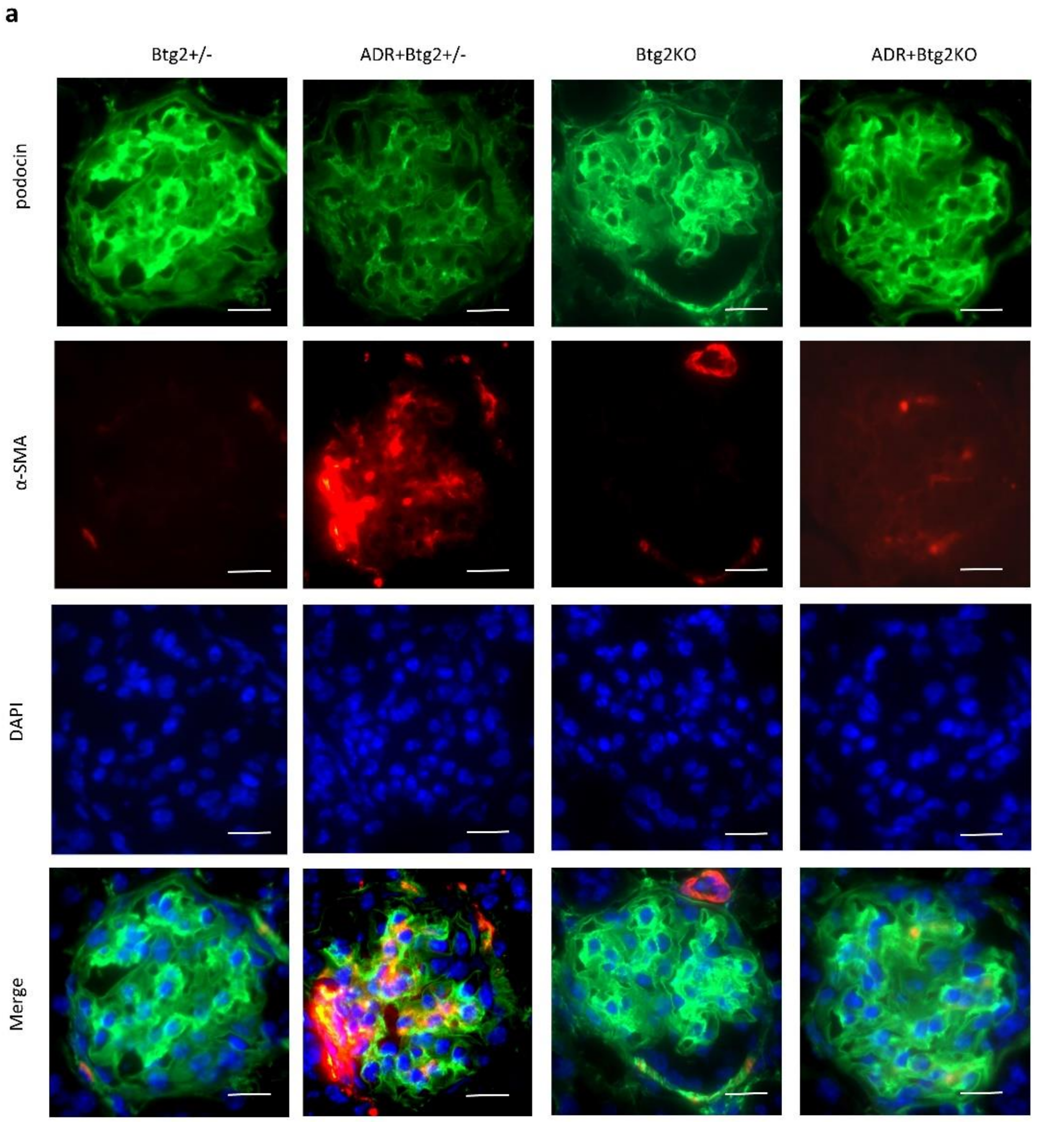

Supplementary figure. 2 (a) Two-color immunofluorescence for colocalization of podocin and α-SMA in Btg2KO and Btg2+/- mice with or without ADR injection. Scale bar =50 μm.

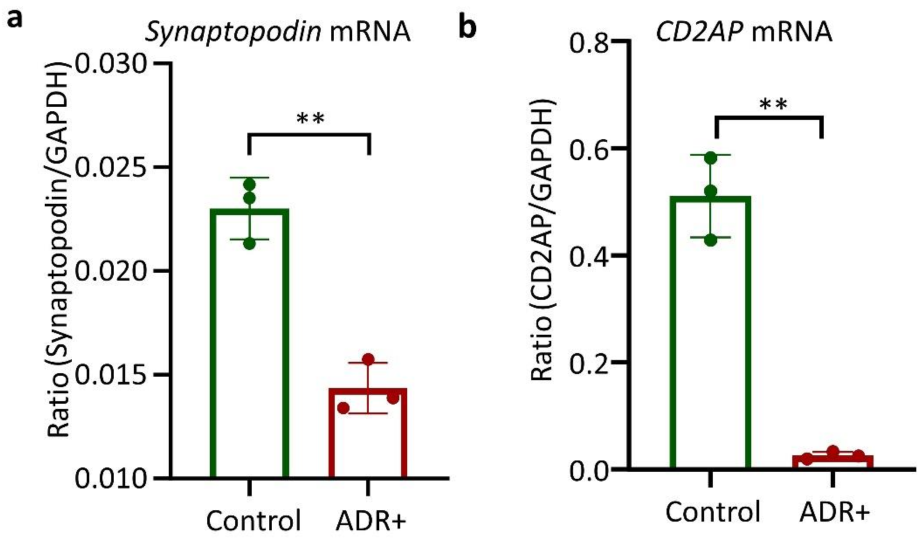

Supplementary Figure 3. (a-b) The mRNA levels of *synaptopodin* and *CD2AP* after ADR injection were determined by RT-qPCR. Data are expressed as the mean±SD(n=6).**P<0.01.ADR=Adriamycin.

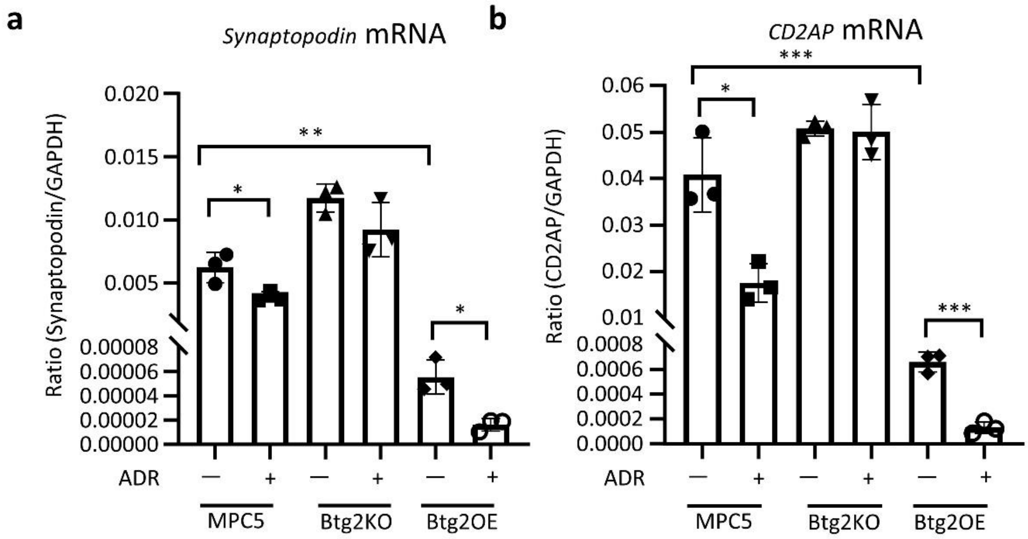

Supplementary Figure 4. (a-b) The mRNA levels of *synaptopodin* and *CD2AP* in both the Btg2 knockout MPC5 sub-cell line and the Btg2-overexpression MPC5 sub-cell line, with or without ADR induction, determined by RT-qPCR. Data are expressed as the mean±SD (n=3).*P<0.05, **P<0.01, ***P<0.001. ADR, Adriamycin; Btg2KO, MPC5 cells with Btg2 knockout; Btg2OE, MPC5 cells overexpressing Btg2.

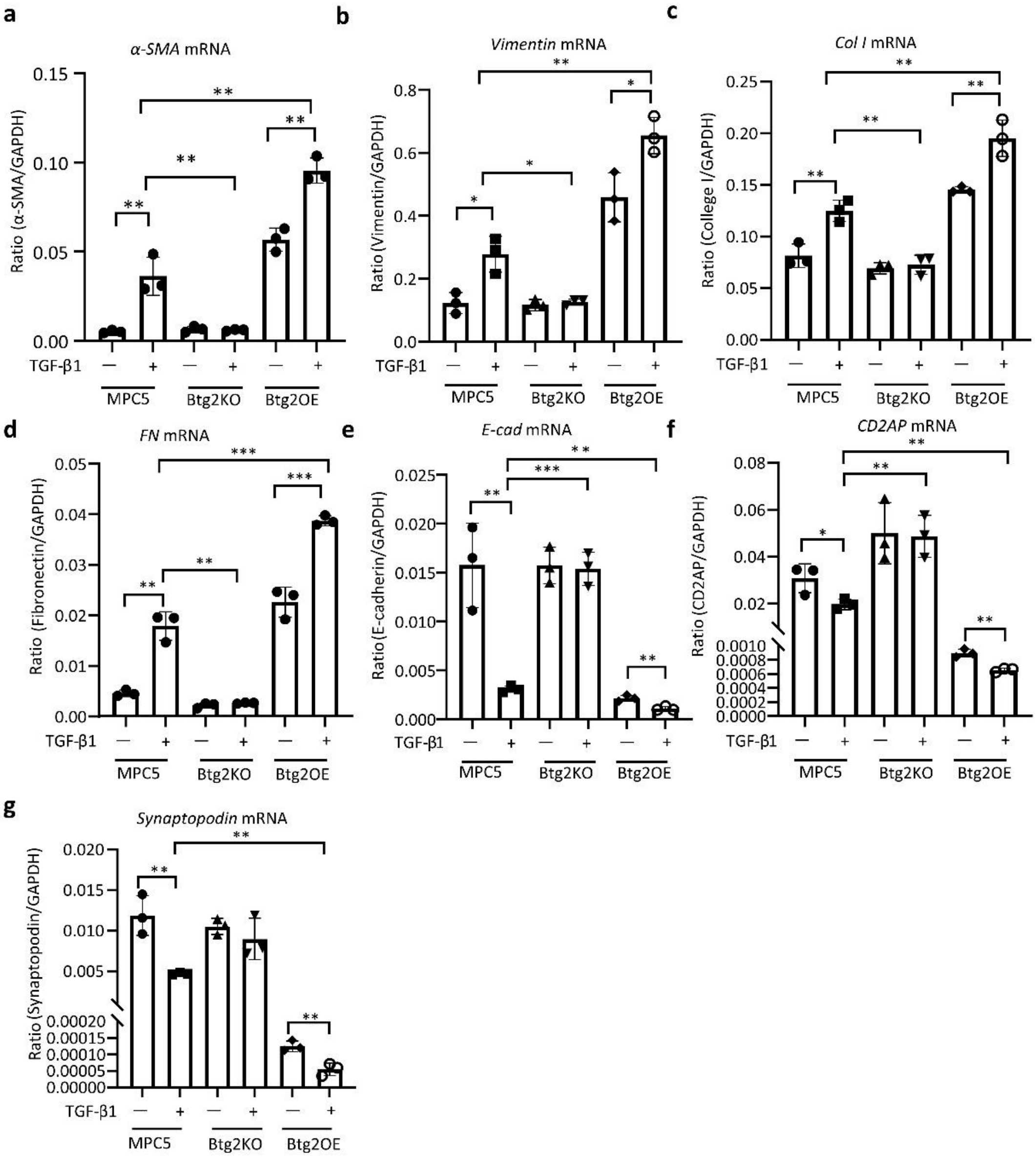

Supplementary Figure 5. (a-g) The mRNA levels of *α-SMA, vimentin, Collagen1*, *fibronectin, E-cadherin, synaptopodin,* and *CD2AP* in both the Btg2 knockout MPC5 sub-cell line and the Btg2-overexpression MPC5 sub-cell line, with or without TGF-β1 induction, determined by RT-qPCR. Data are expressed as the mean±SD (n=3).**P*<0.05, ***P*<0.01, ****P*<0.001. Btg2KO, MPC5 cells with Btg2 knockout; Btg2OE, MPC5 cells overexpressing Btg2; FN, fibronectin; Vim, vimentin; Col1, collagen1; E-cad, E-cadherin.

Supplementary Table 1 Patient's information

Number	pathology number	Pathological diagnosis	Age (year)	Gender	Height (cm)	Weight (kg)	Blood pressure (mmHg)	24-hour urine protein quantification	serum creatinine	Urea nitrogen(mmol/L)	Uric acid(mmol/L)	Cystatin C (mg/L)	Plasma albumin	Plasma globulin	Triglyceride(mol/L)	Cholesterol (mmol)	Low-density lipoprotein(mmol/L)	High-density lipoprotein(mmol/L)	Lipoprotein a(g/L)	Aspartate Transaminase(U/L)	Aspartate aminotransferase(U/L)	Bilirubin(umol)
1	KB2303568	FSGS	18	Male	171	74.8	134/86	25342.5	105	5.57	571	1.56	23.9	27.8	1.96	5.75	1.94	2.86	2.25	40	65	6.2
2	KB2300653	FSGS	14	Female	160	53	160/53	9126.75	63	3	368	1.13	25.6	25.6	3.55	10.38	7.95	1.73	2.12	15	13	6.1
3	KB224865	FSGS	58	Female	148	57	154/83	3885.2	61	12.3	283	1.17	19	21.1	3.45	12.8	4.45	1.95	Not checked	51	50	< 3.4
4	KB224811	FSGS	43	Male	160	60	141/89	104.55	93	5.2	286	1.44	45.6	21.3	1.62	6.43	3.77	2.35	2.25	31	85	15.5
5	KB2230414	Podocyte nephropathy, FSGS not excluded	20	Female	161	60	110/80	8687.7	76	5.13	383	0.8	45	14.5	2.88	14.62	8.71	2.78	Not checked	97	131	6.3
6	KB2229914	Podocyte nephropathy, FSGS not excluded	14	Male	165	60.5	151/84	11129.5	97	5.95	531	1.46	19	31	2.08	11.92	7.89	2.12	2.06	21	9	4.1
7	KB2228550	FSGS with IgA deposition	34	Male	175	105	98/62	22878.6	90	3.67	539	0.83	14.7	31.8	13.92	11.65	5.04	1.49	1.86	22	23	4.4
8	KB222595	FSGS	67	Male	158	75	165/92	5599.02	108	6.9	420	1.43	17.2	27.2	3.86	9.89	Not checked	Not checked	Not checked	18	10	< 3.4
9	KB2218599	FSGS	54	Male	165	59	178/103	14157.6	363	13.0	477	3.7	18.6	26.9	1.51	5.08	3.74	0.84	0.98	20	9	< 3.4
10	2347020090	FSGS	38	Male	170	85	150/97	9859.77	134	11.51	385	3.26	18.8	28.8	1.5	3.96	1.52	2.2	1.65	32	68	10
11	b202300563	Right renal clear cell carcinoma	56	Female	150	53	124/68	Not checked	61	4.2	259	Not checked	42.6	28.7	Not checked	Not checked	Not checked	Not checked	Not checked	26	26	11.8
12	b202302289	Interstitial malignancy of the left kidney	45	Female	158	50	151/93	Not checked	88	5.69	262	Not checked	41.6	32.2	Not checked	Not checked	Not checked	Not checked	Not checked	20	10	12.7

13	b202216678	Left renal clear cell carcinoma	75	Female	158	65	164/86	Not checked	47	5.44	258	0.86	42.2	34.5	Not checked	Not checked	Not checked	Not checked	Not checked	28	25	9.5
14	b202211288	Right renal clear cell carcinoma	59	Male	176	80	160/93	Not checked	83	5.00	334	1.04	46.7	28.6	Not checked	Not checked	Not checked	Not checked	Not checked	22	23	15.0
15	b202210163	Right renal clear cell carcinoma	74	Male	170	70	110/75	Not checked	109	4.68	314	1.29	42.4	31.0	Not checked	Not checked	Not checked	Not checked	Not checked	24	14	10.3
16	b202302720	Left renal clear cell carcinoma	68	Female	164	42	92/68	Not checked	56	5.13	151	1.11	34.1	29.7	Not checked	Not checked	Not checked	Not checked	Not checked	32	40	11.9
17	b202214114	Left renal clear cell carcinoma with infection and necrosis	56	Female	159	50	124/76	Not checked	69	5.60	253	1.06	42.6	29.9	Not checked	Not checked	Not checked	Not checked	Not checked	20	16	10.3
18	b202209221	Right renal clear cell carcinoma	68	Male	165	62	151/75	Not checked	101	8.40	382	1.24	48.1	24.5	Not checked	Not checked	Not checked	Not checked	Not checked	15	13	7.6
19	b202216220	Right renal clear cell carcinoma	52	Female	150	44	106/73	Not checked	60	5.61	235	0.78	45.3	30.9	Not checked	Not checked	Not checked	Not checked	Not checked	21	17	5.1
20	b202210033	Left renal clear cell carcinoma	63	Male	167	63	116/72	Not checked	151	5.37	404	1.59	43.2	32.1	Not checked	Not checked	Not checked	Not checked	Not checked	43.2	32.1	10.4

Note: The diagnosis of FSGS is mainly determined by renal pathological findings. FSGS refers to a pathological form of the kidney, not a disease, characterized by focal glomerular sclerosis under light microscopy (increased mesangial stroma with capillary cavity occlusion, sclerosis, hyalinosis, foam cells, segmental scarring, balloon adhesion). The diagnosis of renal cancer also depends on renal pathology.

Supplementary Table 2. List of antibodies used in this study.

Antibody	Company	Catalog No.	Application
	**Primary antibody**		
Rabbit anti-Btg2	Bioss	Bs-0031R	WB,IF,IHC,
Mouse anti-Btg2	Santa Cruz	Sc-517187	IF,Co-IP
Rabbit anti-Podocin	Boster	BA0290	WB,IF
Rabbit anti-α-smooth muscle actin	Proteintech	14395-1	IHC,WB,IF
Rabbit anti-Fibronectin	Abmart	T59537	IHC,WB,IF
Rabbit anti-Collagen I	Abmart	TA7001	IHC,WB,IF
Rabbit anti-vimentin	Proteintech	10366-1-AP	IHC,WB,IF
Rabbit anti-E-cadherin	Proteintech	20874-1-AP	IHC,WB,IF
Rabbit anti-GAPDH	Abways	AB0037	WB
Rabbit anti-HISTAH3A	Sangon Biotech	D124055	WB
Rabbit anti-Phospho-Smad3	Cell Signaling	C25A9	WB,IHC
Rabbit anti-Smad3	Cell Signaling	C67H9	WB
Rabbit anti-Phospho-Smad2	Cell Signaling	5339	WB
Rabbit anti-Smad2	Cell Signaling	18338	WB
Rabbit anti-TGF-β1	Boster	BA0290	WB,IHC
	**Secondary antibody**		
Goat anti-Rabbit IgG (H+L) Cross-Adsorbed Secondary Antibody, Alexa Fluor™ 488	Thermo Fisher Scientific	A-11008	IF
Goat anti-Rabbit IgG (H+L) Highly Cross-Adsorbed Secondary Antibody, Alexa Fluor™ Plus 680	Thermo Fisher Scientific	A32734	WB
Goat anti-Rabbit IgG (H+L) Highly Cross-Adsorbed Secondary Antibody, Alexa Fluor™ Plus 555	Thermo Fisher Scientific	A32732	IF
Goat anti-Mouse IgG (H+L) Highly Cross-Adsorbed Secondary Antibody, Alexa Fluor™ Plus 555	Thermo Fisher Scientific	A32727	IF

Note: WB, Western blot. IHC, immunohistochemistry. IF, immunostaining. Co-IP, Co-Immunoprecipitation.

Supplementary Table 3. Primers used in RT-PCR

Gene Name	Primer sequence 5'-3'	Product Length
**GAPDH**	F: CGGAGTCAACGGATTTGGTCGTAT	24
	R: AGCCTTCTCCATGGTGGTGAAGAC	24
**BTG2**	F: ATGAGCCACGGGAAGAGAAC	20
	R: GCCCTACTGAAAACCTTGAGTC	22
**Podocin**	F: GACCAGAGGAAGGCATCAAGC	21
	R: GCACAACCTTTATGCAGAACCAG	23
**Nephrin**	F: ATGGGAGCTAAGGAAGCCACA	21
	R: GATGGAGAGGATTACGCTGGG	21
**Synaptopodin**	F CTCAGTGACTCTGATTCCAG	20
	R TGTGCCTCATCTAACTCCAG	20
**CD2AP**	F: CCTCCTCCTGCAAAAGGTCC	20
	R: GCTTTGGGTACACTGCTCCA	20
**α-SMA**	F:CCCAGACATCAGGGAGTAATGG	22
	R:TCTATCGGATACTTCAGCGTCA	22
**Fibronectin**	F:ATGTGGACCCCTCCTGATAGT	20
	R:GCCCAGTGATTTCAGCAAAGG	21
**Collagen I**	F:ATCCAACGAGATCGAGCTCA	20
	R:AAGGGAGCCACATCGATGAT	20
**Vimentin**	F:GCCAGATGCGTGAGATGGAA	20
	R:ATTCACGAAGGTGACGAGCC	20
**E-Cadherin**	F:GAAGGCTTGAGCACAACAGC	20
	R:CCCTGATACGTGCTTGGGTT	20
